# Uncertainty Estimation of the Dose Rate in Real-Time Applications Using Gaussian Process Regression

**DOI:** 10.3390/s20102884

**Published:** 2020-05-19

**Authors:** Jinhwan Kim, Kyung Taek Lim, Kyeongjin Park, Yewon Kim, Gyuseong Cho

**Affiliations:** 1 Department of Nuclear and Quantum Engineering, Korea Advanced Institute of Science and Technology, 291, Daehak-ro, Yuseong-gu, Daejeon 34141, Korea; kjhwan0205@kaist.ac.kr (J.K.); kl2548@kaist.ac.kr (K.T.L.); myesens@kaist.ac.kr (K.P.); 2 The Center for Nuclear Nonproliferation Strategy and Technology, Korea Institute of Nuclear Nonproliferation and Control, 1534 Yuseong-daero, Yuseong-gu, Daejeon 34054, Korea; yewonkim@kinac.re.kr

**Keywords:** spectrum-to-dose conversion operator, G(E) function, gaussian process regression, dose rate uncertainty, real-time dosimetry, operational quantities

## Abstract

Major standard organizations have addressed the issue of reporting uncertainties in dose rate estimations. There are, however, challenges in estimating uncertainties when the radiation environment is considered, especially in real-time dosimetry. This study reports on the implementation of Gaussian process regression based on a spectrum-to-dose conversion operator (i.e., G(E) function), the aim of which is to deal with uncertainty in dose rate estimation based on various irradiation geometries. Results show that the proposed approach provides the dose rate estimation as a probability distribution in a single measurement, thereby increasing its real-time applications. In particular, under various irradiation geometries, the mean values of the dose rate were closer to the true values than the point estimates calculated by a G(E) function obtained from the anterior–posterior irradiation geometry that is intended to provide conservative estimates. In most cases, the 95% confidence intervals of uncertainties included those conservative estimates and the true values over the range of 50–3000 keV. The proposed method, therefore, not only conforms to the concept of operational quantities (i.e., conservative estimates) but also provides more reliable results.

## 1. Introduction

The concepts of equivalent dose and effective dose were first introduced by the International Commission on Radiological Protection (ICRP) in order to provide recommendations and guidelines for the protection of people and the environment in an integrated manner in all exposure situations [1,2,3]. However, given that these concepts are not measurable quantities, the International Commission on Radiological Units and Measurements (ICRU) defined a few measurable operational quantities to establish convenient and appropriate evaluations of an equivalent and effective dose [4,5]. In cases of strongly penetrating radiations, such as gamma rays and neutrons, an adequate operational quantity for monitoring a specific area is defined by the ambient dose equivalent H*(10) (hereafter referred to as "ambient dose rate" and used interchangeably with the term "dose rate"). The ICRU defined the ambient dose rate as, "The dose equivalent at a point in a radiation field that would be produced by a corresponding expanded and aligned field in the ICRU sphere at a depth of 10 mm on the radius vector opposing the direction of the aligned field."

The response of the ambient dose rate is highly dependent on photon energy and the angle of radiation incidence. Therefore, the requirement of IEC 60846:2009 advises that the relative response of the dose rate to the reference radiation (e.g., Cs-137) within the combined rate range of photon energy and the angle of incidence shall be between 0.6 and 1.4 [6]. Intending to minimize the effect of photon energy on dose rate response and to achieve more accurate estimates of the dose rate, the use of scintillation detectors is a way to make the response less sensitive to radiation energy by obtaining an energy spectrum. The G(E) function is a typical example of conversion from the energy spectrum to dose rate [7,8,9,10,11,12]; the measured spectrum is directly converted into dose rate without applying stripping or unfolding methods [13,14,15]. This method is, therefore, often adopted for real-time dose measurement. For the estimation of the G(E) function, the most conservative direction of irradiation, anterior posterior, is typically assumed rather than those in other idealized geometries, such as rotational and isotropic ones. However, in a real contaminated environment, the irradiation direction of photons entirely depends on typically unknown source distributions. Although isotropic or rotational geometry approximate certain real irradiation conditions [9,16], these are not the same as the idealized ones [17]. In addition, since the response of dosimetry is normalized to the ambient dose rate under one of the geometric conditions, most errors in dose rate estimation primarily arise from the calculation of the dose conversion operator. From a safety standpoint, it is necessary to provide conservative dose rate estimates. In this respect, an alternative is the use of the maximum value for the dose conversion operator, which would be similarly produced by various irradiation geometries, as already proposed [18]. However, it is often more important to report the best estimate and the best evaluation of dose rate uncertainty that includes a conservative estimate. Therefore, a different approach would be preferable to ensure that the dose rate is presented with the best estimate of the mean and its expending uncertainty (i.e., 1.96 standard deviations) for real-time applications.

**Figure 1:**
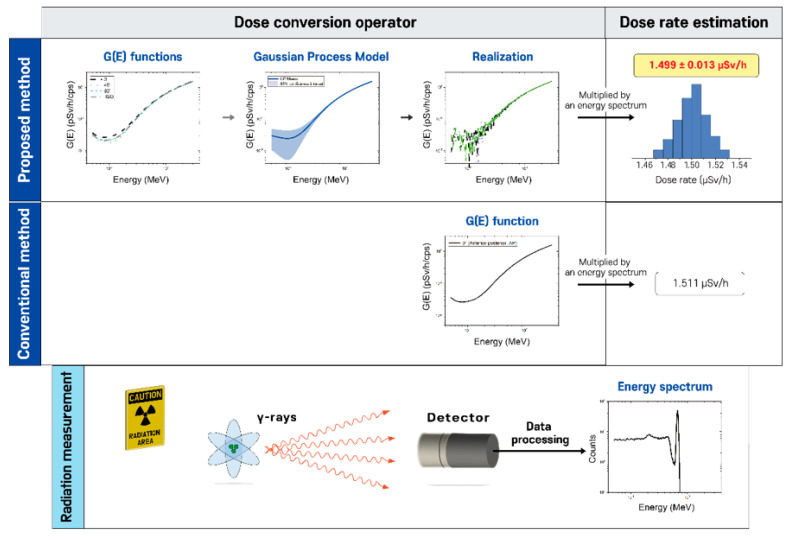
Illustration of the proposed concept of dealing with uncertainty in dose rate estimation compared with the conventional method.

This study presents a new spectrum-to-dose conversion operator concept, called $G{\left(E\right)}_{GPR}$ functions, which are G(E) functions based on Gaussian process (GP) regression that account for the relative response to radiation energy and direction of radiation incidence in order to deal with uncertainty in dose rate estimation. A GP model can be constructed using all the data points of G(E) functions determined under various irradiation geometries, e.g., the angles of incidence of 0°, 45°, and 90°, and isotropic geometry. Then, a set of $G{\left(E\right)}_{GPR}$ functions can be obtained through independent realizations (or equivalently, sample path) of the GP model, where each realization defines a conversion factor for every possible energy step. Lastly, the obtained $G{\left(E\right)}_{GPR}$ functions are multiplied by an observed spectrum to make it possible to estimate the mean dose rate value and its associated uncertainty. Figure 1 illustrates the proposed concept in comparison with the conventional method. This paper presents simulation results that demonstrate the behavior and performance of the proposed approach. It is worth noting that although this study focuses on the ambient dose rate, this method can be applied to any dosimetric quantity (e.g., air kerma) if the target quantity is defined as a function of radiation energy.

## 2. Materials and Methods

### 2.1. G(E) Function

A general description of the G(E) function in terms of the dose conversion coefficient $h\left(E_{0}\right)$ at mono-energy $E_{0}$ and the response function of a detector $R\left(E,E_{0}\right)$, which represents the photon of energy $E_{0}$ depositing energy E into the detector, can be expressed as

(1) $$h\left(E_{0}\right)=\int_{E_{min}}^{E_{max}}R\left(E,E_{0}\right)G\left(E\right)dE,$$

where $E_{min}$ and $E_{max}$ are the minimum and maximum detectable energies deposited in the detector, respectively. The total dose rate (D) in multi-energy radiation conditions can, therefore, be represented as

(2) $$D=\underset{i}{\sum }\emptyset \left(E_{i}\right)h\left(E_{i}\right)\;=\underset{i}{\sum }\emptyset \left(E_{i}\right)\int_{E_{min}}^{E_{max}}R\left(E,E_{i}\right)G\left(E\right)dE\;=\int_{E_{min}}^{E_{max}}\underset{i}{\sum }\emptyset \left(E_{i}\right)R\left(E,E_{i}\right)G\left(E\right)dE\;=\int_{E_{min}}^{E_{max}}M\left(E\right)G\left(E\right)dE.$$

where $\emptyset \left(E_{i}\right)$ is the fluence rate at the energy of $E_{i}$. In real conditions, the integral of continuous energies should be changed to the sum of discrete energies (or equivalently, the number of channels  N; in this case, N = 869).

(3) $$D=\overset{N}{\underset{i=1}{\sum }}M\left(E_{i}\right)G\left(E_{i}\right)$$

Consequently, the total dose rate can be directly estimated using the G(E) function and a measured spectrum. According to related studies [7,8,9,11], the G(E) function can be expressed as

(4) $$G\left(E\right)=\overset{K_{max}}{\underset{K=1}{\sum }}A\left(K\right){\left(log\left(E\right)\right)}^{K-M-1},$$

where A(K) is a parameter, $K_{max}$ is the number of terms, and M is constant? The values for $K_{max}$ and M were set to 7 and 0, respectively. It should be noted that the optimization of these parameters is not the main concern of this study. Dose rate can be represented by combining Equations (3) and (4):

(5) $$D=\overset{N}{\underset{i=1}{\sum }}M\left(E_{i}\right)\overset{K_{max}}{\underset{K=1}{\sum }}A\left(K\right){\left(\log\left(E_{i}\right)\right)}^{K-M-1}.$$

To compute A(K), it is required to obtain spectra with known mono or multiple energies and the corresponding dose rates. The availability of energy sources limits actual experiments; however, Monte Carlo simulations allow for the use of any energy, so corresponding dose rates can be calculated, given that detector geometry has been properly defined. Finally, A(K) were obtained using the gradient descent method [11].

### 2.2. GP Regression

This section briefly introduces the concept of GP regression employed for implementation purposes to deal with uncertainty in dose rate estimation. More details can be found in [19,20].

The GP is expressed as a distribution over functions for which any finite subset of variables has a joint multivariate Gaussian distribution. Since the GP is described by Gaussian distribution, it is parameterized by its mean function m(x) and positive definite covariance function k(x, x′), also known as a kernel function:

(6) f(x)~GP(m(x),k(x, x′))

Typically, m(x) is set to 0 to avoid expensive computations in posterior distribution and make inferences only via the kernel function. The kernel function takes two indices x and x′ and returns their corresponding modeled covariance. By choosing an appropriate kernel function, it is possible to incorporate assumptions such as smoothness and likely patterns that are expected in the data. A popular choice of the kernel is the radial basis function kernel, where two points are exponentially correlated, depending on the distance between them.

The main assumption in GP modeling is that output y is an observation of f(x) that has been corrupted by Gaussian noise ϵ:

(7) $$y=f\left(x\right)+\varepsilon ,\;\varepsilon \;\sim \;N\left(0,\sigma_{\varepsilon }^{2}\right)$$

where noise term ϵ is assumed independent and identically distributed with zero means. Hence f(x) is a latent variable whose posterior distribution will be inferred after observing new samples at various locations in the domain. The resultant inference is called GP regression.

Suppose training outputs $y_{t}$ have been observed and predictions for test outputs $f_{*}$ have been made. They then follow a joint normal distribution:

(8) $$\left[\begin{array}{c} y_{t}\\ f_{*} \end{array}\right]\sim N\left(0,\left[\begin{array}{cc} K\left(X_{t},X_{t}\right)+\sigma_{\varepsilon }^{2}Ι & K\left(X_{t},X_{*}\right)\\ K\left(X_{*},X_{t}\right) & K\left(X_{*},X_{*}\right) \end{array}\right]\right)$$

where $X_{t}$ and $X_{*}$ are the design matrices for training and test data, respectively. $K\left(X_{t},X_{t}\right)$ represents the covariance matrix between all points observed so far in the training data, which is similarly true for other covariance matrices of $\;K\left(X_{t},X_{*}\right)$, $K\left(X_{*},X_{t}\right)$, and $\;K\left(X_{*},X_{*}\right)$. Ι  is an identity matrix whose diagonal elements and off-diagonal elements are 1 and 0, respectively? Conditioning $f_{*}$ on the observation ${\;y}_{t}\;p\left(f_{*}{|X}_{t},y_{t},X_{*}\right),$ the predictive distribution of test points with respect to the mean and covariance matrix can be written as:

(9) $$m_{t}\left(x\right)=K\left(x,X_{t}\right){\left[K\left(X_{t},X_{t}\right)+\sigma_{\varepsilon }^{2}Ι\right]}^{-1}y_{t}$$

(10) $$K_{t}\left(x,x\prime \right)=K\left(x,x\prime \right)-K\left(x,X_{t}\right){\left[K\left(X_{t},X_{t}\right)+\sigma_{\varepsilon }^{2}Ι\right]}^{-1}K\left(X_{t},x\prime \right)$$

Consequently, the estimation of the posterior mean and covariance are involved in calculating four different covariance matrices. 

### 2.3. Monte Carlo Modeling and Simulation

**Figure 2:**
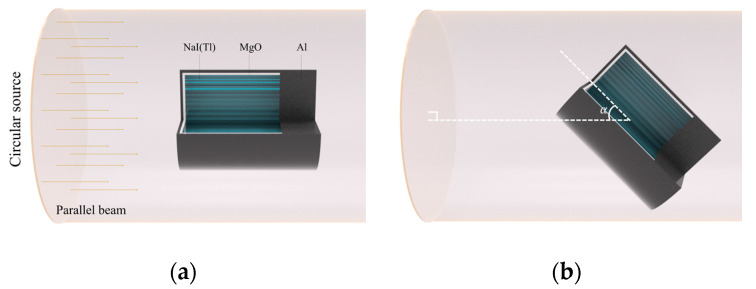
Schematic of the calculation geometry defined for MCNP6 simulations. (**a**) A parallel photon beam was irradiated on an NaI(Tl) detector. (**b**) The directions of the detector were rotated against the circular source by specific angle α. A 20 mm-thick aluminum disk was positioned on the back of the crystal to consider the scattering in a photomultiplier tube.

A Monte Carlo N-Particle Transport Code (MCNP6) [21] was used to validate the proposed method. A schematic of the MCNP6 model used for simulations is illustrated in Figure 2. The 5.08 × 5.08 cm (diameter × height) NaI(Tl) crystal with a density of 3.6 g cm^−3^ was covered by a MgO reflector with a density of 2 g cm^−3^, which was surrounded by aluminum with a density of 2.7 g cm^−3^. A 20-mm-thick aluminum plate was placed behind the crystal to mimic a phenomenon where photons are scattered or backscattered in a photomultiplier tube [22]. This is a reasonable assumption because it considers the scattered effects of the photomultiplier tube on a spectrum as the angle of irradiation direction changes. A parallel beam of photons distributed over a circular source was irradiated on the NaI(Tl) detector. In order to obtain $G{\left(E\right)}_{g}$ functions under various directions of irradiation, the directions of the detector were rotated against the circular source by specific angle α (i.e., 0°, 45°, and 90°), where subscript g is the irradiation geometry that determines the G(E) function. For isotropic geometry, the detector was centered inside the spherical source, emitting fully isotropic irradiation of photons.

Since an actual spectrum is influenced by the broadening effect due to the statistical variation of the scintillation light signals and various electronic sources of noise, a simulated spectrum must be modified to accommodate such effects. This can be regarded as a convolution process of an ideal spectrum with the kernel of a broadening filter. A Gaussian-energy broadening filter is often applied, meaning that a delta function type of peak becomes a Gaussian function with full-width at half-maximum value (FWHM = 2.36 × sigma). In the MCNP, a non-linear function with three parameters regarding FWHM is specified to apply broadening effects on the ideal spectrum. The optimal values of parameters obtainable from measured spectra were found using a genetic algorithm [23].

## 3. Results

### 3.1. G(E) Functions for Idealized Irradiation Geometires

**Figure 3:**
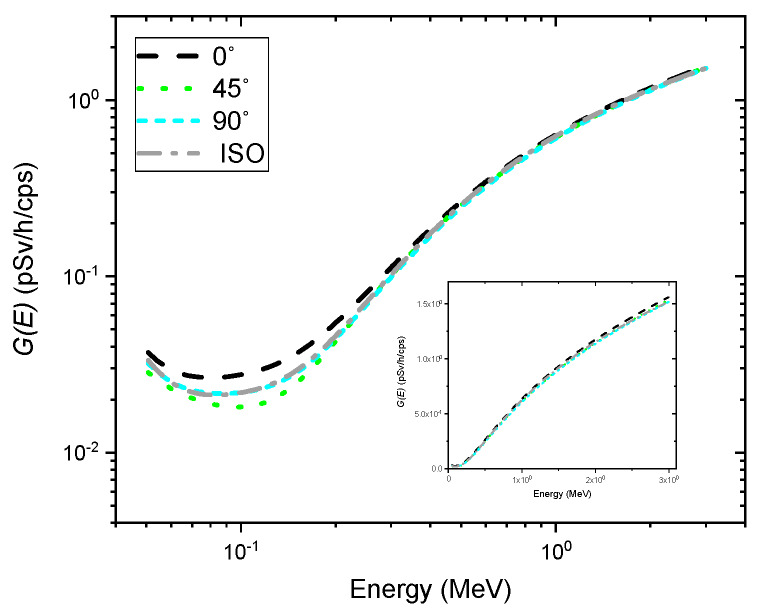
Spectrum-to-dose conversion operator (i.e., G(E) functions) for the angles of incidence of 0° (black dashed line), 45° (green dotted line), and 90° (cyan short-dashed line), and isotropic geometry (gray dash-dotted line). The inset shows the same graph on a linear scale.

Figure 3 shows the determined G(E) functions of the NaI (Tl) detector as a function of energy deposited in the crystal under four different irradiation geometries. As expected, the $G{\left(E\right)}_{0{}^{\circ}}$ function, i.e., G(E) function for the angle of incidence of 0°, tended to yield higher values over the entire energy range than those for other directions of photons with respect to the detector axis. Additionally, there were relatively small differences between the values of G(E) functions for energies above 200 keV, showing good agreement with previous results [9,12]. This could be ascribed to the fact that the energy deposition of relatively high energy depends primarily on the volume of the crystal. On the other hand, the values of the G(E) functions obtained from different types of irradiation geometry tended to relatively disperse, especially for energies below 200 keV. This is because the photons in that energy range have high interaction probabilities with the crystal, so the energy deposition in the crystal becomes proportional to the projected area of the crystal incident surface. These results suggest that the estimated dose rate may drift from the true value, especially in the low energy range, depending on the G(E) functions calculated by different types of irradiation geometry.

### 3.2. G(E) Functions Using GP Regression

**Figure 4:**
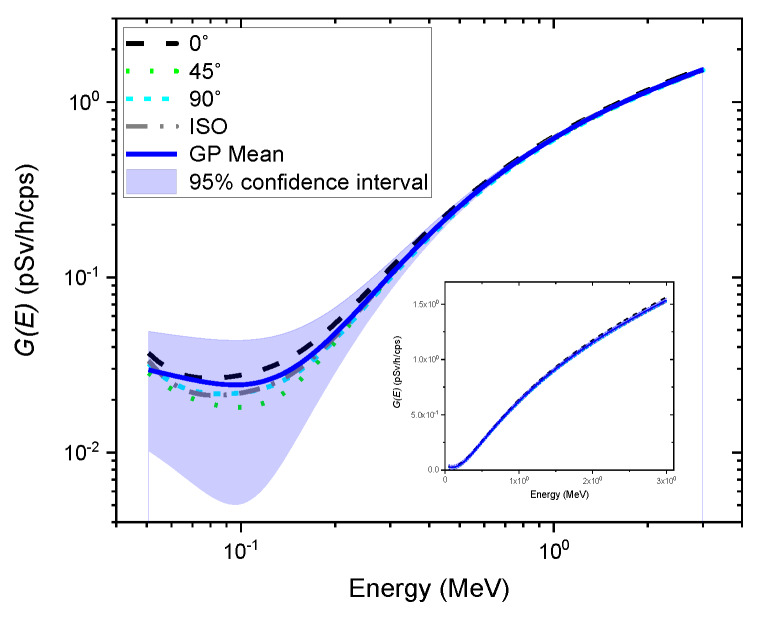
Gaussian process (GP) regression using the data from the previously-determined G(E) functions under the angles of incidence of 0° (black dashed line), 45° (green dotted line), and 90° (cyan short-dashed line), and isotropic geometry (gray dash-dotted line). The blue solid line represents the mean of the GP model. The blue shaded area denotes a 95% confidence interval. The previously determined G(E) functions are also illustrated for comparison. The inset shows the same graph on a linear scale.

Figure 4 shows the posterior mean of the GP model as well as its probabilistic nature in the form of a 95% confidence interval using the data from previously-determined G(E) functions; the G(E) functions are also illustrated in this figure for comparison. The result shows that the GP model no longer has a single value for energy but a distribution (i.e., Gaussian distribution) indexed by energy. In addition, the entire data points of G(E) functions determined under different types of irradiation geometry were found inside the 95% confidence region of the posterior. In particular, the relative vertical width of the confidence region with regard to the mean value tended to increase as it moved to the low energy range, especially for energies below 200 keV, to account for variations induced by radiation energy and direction of radiation incidence. It should be noted that the absolute values of the confidence region obtained from the GP model over the entire energy range are almost similar.

**Figure 5:**
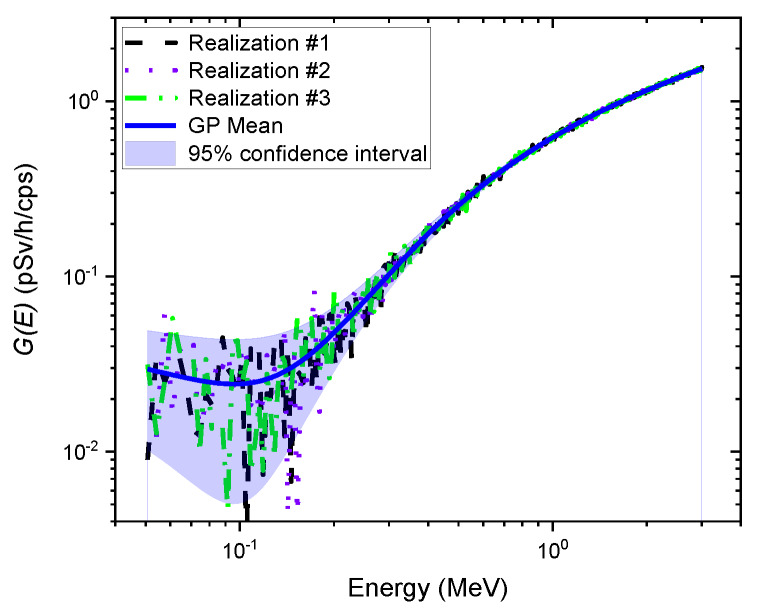
Example of three independent realization functions (i.e., $G{\left(E\right)}_{GPR}$ functions) randomly sampled from the GP model. The solid blue line represents the mean of the GP model. The blue shaded area denotes the 95% confidence interval.

Figure 5 shows an example of independent realization functions (i.e., $G{\left(E\right)}_{GPR}$ functions) randomly sampled from the GP model. As expected, each $G{\left(E\right)}_{GPR}$ function represented a different path because of the randomness of the stochastic process, fluctuating around the mean of the GP model. This implies that multiple dose rate values can be calculated using the $G{\left(E\right)}_{GPR}$ functions multiplied by the observed spectrum, resulting in not only the best dose rate estimate but also its uncertainty, which might contain the true value. The mean of the GP model was lower than that of the $G{\left(E\right)}_{0{}^{\circ}}$ function, which generally overestimates dose rates, so it nearly coincided with the $G{\left(E\right)}_{ISO}$ function (see Figure 4). That is, the mean dose rate values and the dose rates estimated by the $G{\left(E\right)}_{ISO}$ function might be in good agreement. This result is quite promising because isotropic geometry can be a reasonable assumption for irradiations often received from naturally occurring radioisotopes in homes or the surrounding environments [9,16].

### 3.3. Dose Rate Uncertainty Estimation

**Figure 6:**
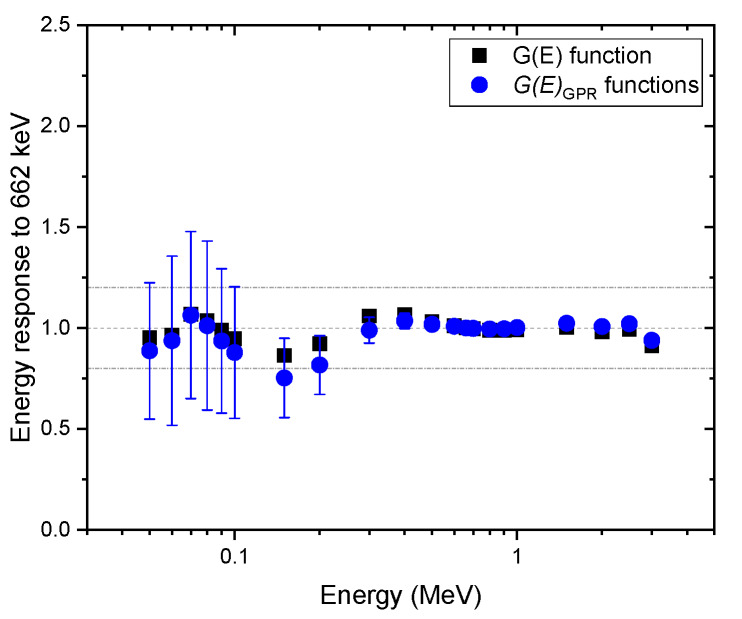
Comparison of energy response normalized to the energy of 622 keV, estimated with $G{\left(E\right)}_{GPR}$ functions, and the $G{\left(E\right)}_{0{}^{\circ}}$ function for the spectra obtained under the angle of incidence of 0°. The error bar shows a 95% confidence interval.

To validate the proposed method, various spectra were obtained for mono-energy over the range of 50–3000 keV at certain intervals with various geometries, e.g., the angles of incidence of 0°, 45°, and 90°, and isotropic geometry. To calculate the uncertainty of the dose rate (i.e., 95% confidence interval), 100 $G{\left(E\right)}_{GPR}$ functions were randomly sampled from the GP model each time. Figure 6 shows a comparison of the energy response normalized to the energy of 622 keV emitted by Cs-137, estimated with $G{\left(E\right)}_{GPR}$ functions and the $G{\left(E\right)}_{0{}^{\circ}}$ function for the spectra obtained at the angle of incidence of 0°. Here, the energy response was calculated by having the ratio of the estimated dose rate to the true dose rate at certain energy divided by the same ratio at the energy of 662 keV. That is, an increase in the value of the energy response suggests that the dose rate is overestimated, or vice versa. As we can see from the figure, the energy responses for the $G{\left(E\right)}_{0{}^{\circ}}$ function were reasonably close to one, which means that the estimated values of the dose rate and true values were in good agreement. This is because the test spectra were acquired under the same condition used for the $G{\left(E\right)}_{0{}^{\circ}}$ function calculation. These results are not as good as those that were reported by previous studies, especially for the low energy range. Nonetheless, it is worth emphasizing that the purpose of this study was to propose a concept that would make it possible to deal with uncertainty existing in the dose rate by taking into account the relative response of radiation energy and the direction of radiation incidence. For the dose rates estimated with $G{\left(E\right)}_{GPR}$ functions, the mean values deviated slightly more from the reference value 0 for energies below 200 keV. The reason is that there might be a discrepancy between the mean values of $G{\left(E\right)}_{GPR}$ functions sampled from the GP model and those from the $G{\left(E\right)}_{0{}^{\circ}}$ function, which is better suited with respect to the test spectra. In contrast, the proposed method was able to provide the uncertainty and the mean of the dose rate. As expected, the relative uncertainty tended to increase with a decrease in energy. In particular, the relative uncertainty increased sharply for energies under 200 keV because the GP model had relatively wide intervals for that energy range. In addition, the 95% confidence interval of relative uncertainty mostly included the true value and the conservative values obtained with the $G{\left(E\right)}_{0{}^{\circ}}$ function.

**Figure 7:**
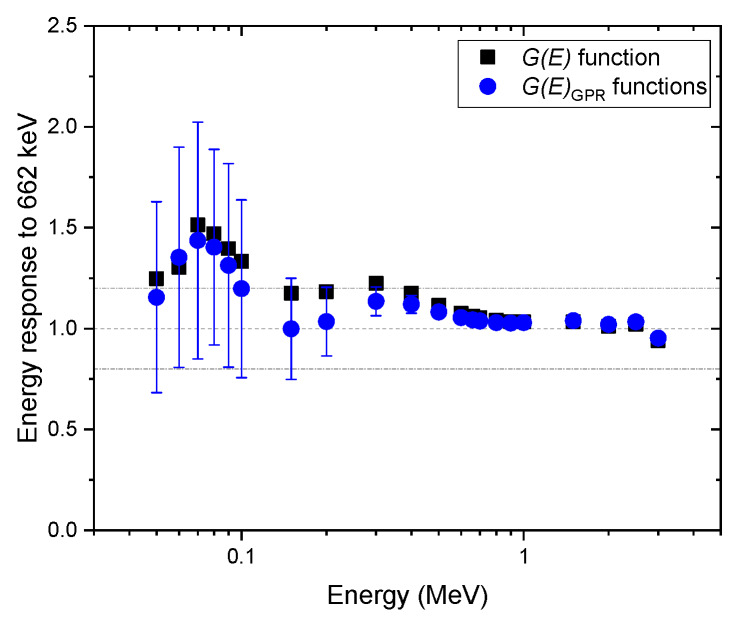
Comparison of energy response normalized to the energy of 622 keV, estimated with $G{\left(E\right)}_{GPR}$ functions, and the $G{\left(E\right)}_{0{}^{\circ}}$ function for the spectra obtained under the angle of incidence of 45°. The error bar shows a 95% confidence interval.

**Figure 8:**
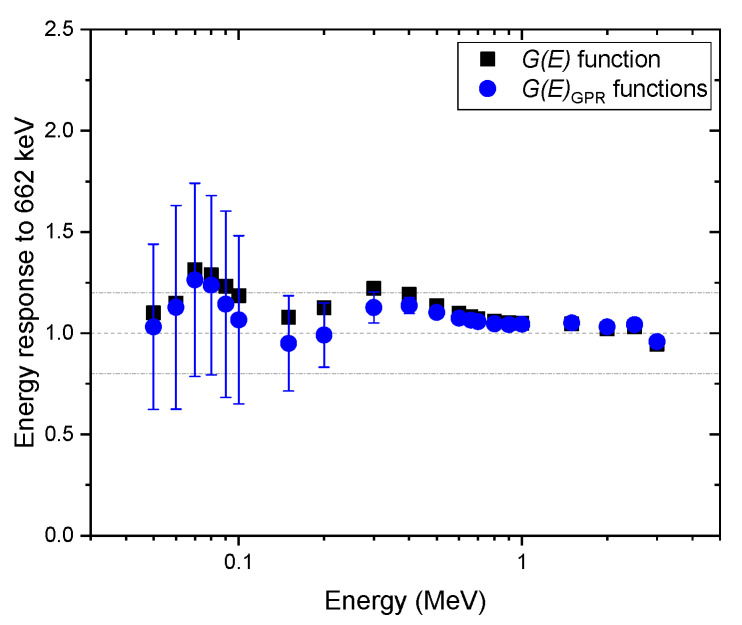
Comparison of energy response normalized to the energy of 622 keV, estimated with $G{\left(E\right)}_{GPR}$ functions, and the $G{\left(E\right)}_{0{}^{\circ}}$ function for the spectra obtained under the angle of incidence of 90°. The error bar shows a 95% confidence interval.

**Figure 9:**
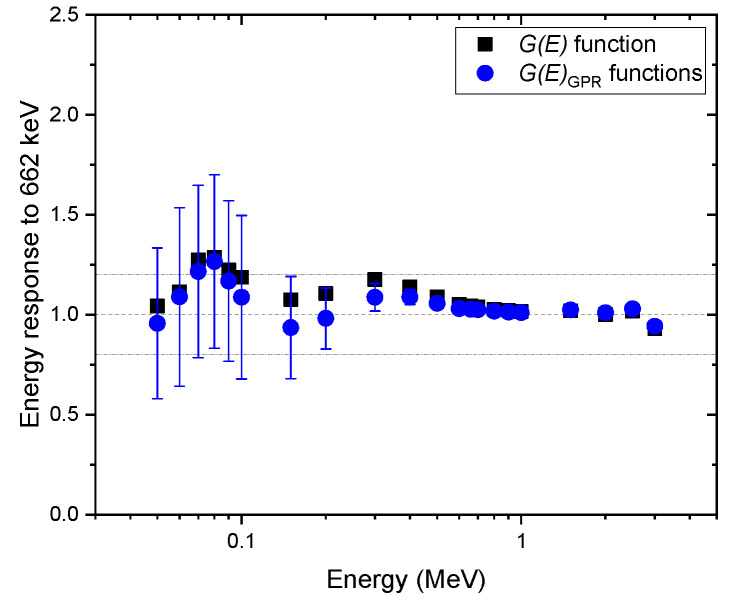
Comparison of energy response normalized to the energy of 622 keV, estimated with $G{\left(E\right)}_{GPR}$ functions, and the $G{\left(E\right)}_{0{}^{\circ}}$ function for the spectra obtained under isotropic geometry. The error bar shows a 95% confidence interval.

Figure 7, Figure 8 and Figure 9 show the same comparison of energy response for the spectra obtained at the angles of incidence of 45° and 90°, and isotropic geometry. Although similar trends were observed in the non-zero angle of incidences, the estimated dose rate values obtained with the $G{\left(E\right)}_{0{}^{\circ}}$ function were overestimated, especially for energies below 600 keV for all geometries in which the test spectra were acquired, which was expected. This shows the reason that the $G{\left(E\right)}_{0{}^{\circ}}$ function is generally used to provide conservative dose estimates. In particular, the dose rate overestimation for the test spectra, assuming that photons were irradiated under the angle of incidence of 45°, is as high as 50% at 70 keV (see Figure 7). This is because the largest projected area of the crystal incident surface is generated at that specific angle, which increases interaction probabilities, especially for photons at low energies. Likewise, the mean values of the dose rate estimate, with $G{\left(E\right)}_{GPR}$ functions, showed similar trends but were less overestimated. Furthermore, the uncertainties of those estimates included not only the conservative values estimated by the $G{\left(E\right)}_{0{}^{\circ}}$ function but also the true values in most situations. The uncertainty calculated by the proposed method is much more reliable than those that provide only a point estimate, because in the real world, it is not possible to know how far an estimate is from the true value.

## 4. Discussion

This paper presented how GP regression can be applied to deal with dose rate uncertainty in real-time applications. The results demonstrated that the proposed approach is much more reliable and robust in comparison with existing methods. For conventional methods, a way to determine uncertainties is the statistical analysis of a series of observations (i.e., Type A uncertainties). In this case, however, they ignore other components of uncertainty determined by scientific judgment based on published data (e.g., G(E)  function). Furthermore, in cases of real-time applications, the estimation of Type A uncertainties is practically impossible, so the uncertainty associated with dose rate can, therefore, not be reported. Although previous studies attempted to estimate dose rate uncertainty, they simply averaged G(E) functions for the angles of incidence of 0° and 90° over the entire energy range and neglected the combined effects of the direction of radiation incidence and photon energy on the detector response [24]. In contrast, this study constructed a GP model that considers the relative responses to various irradiation geometries and energy to provide the mean and its uncertainty for the estimated dose rate based on a single spectrum. In addition, the mean dose rate values were not heavily overestimated under various irradiation geometries, and the 95% confidence interval of uncertainties included the conservative estimates obtained with the $G{\left(E\right)}_{0{}^{\circ}}$ function and true values. An estimated value without a statement about its associated uncertainty is less informative because it does not make it possible to quantify the potential risk arising from radiation exposure and does not indicate the precision of the estimate. Lastly, the calculated uncertainty allows for a quantitative comparison with results reported by other investigators, which enables its assessment.

## 5. Conclusions

This work presented a new method for dose rate estimation that uses GP regression. The presented results confirmed that numerous $G{\left(E\right)}_{GPR}$ functions that account for the relative responses to radiation energy and irradiation directions could be randomly sampled from a GP model, making it possible to deal with uncertainty in dose rate estimation for real-time applications. While the conventional method overestimates the dose rate by as much as 50% under different irradiation geometries, the mean values of the dose rate estimated with $G{\left(E\right)}_{GPR}$ functions were closer to the true value. Furthermore, the overestimated values obtained with the $G{\left(E\right)}_{0{}^{\circ}}$ function and the true values were mostly found within the 95% confidence interval of uncertainty. Therefore, the proposed method conforms to the concept of operational quantities present in conservative estimates and provides a more reliable dose rate estimation.
